# Upregulated Expression of Integrin α1 in Mesangial Cells and Integrin α3 and Vimentin in Podocytes of *Col4a3*-Null (Alport) Mice

**DOI:** 10.1371/journal.pone.0050745

**Published:** 2012-12-07

**Authors:** Brooke M. Steenhard, Roberto Vanacore, David Friedman, Adrian Zelenchuk, Larysa Stroganova, Kathryn Isom, Patricia L. St. John, Billy G. Hudson, Dale R. Abrahamson

**Affiliations:** 1 Department of Anatomy and Cell Biology, University of Kansas Medical Center, Kansas City, Kansas, United States of America; 2 Kidney Institute, University of Kansas Medical Center, Kansas City, Kansas, United States of America; 3 Department of Medicine, Vanderbilt University Medical Center, Nashville, Tennessee, United States of America; 4 Department of Biochemistry, Vanderbilt University Medical Center, Nashville, Tennessee, United States of America; National Cancer Institute, United States of America

## Abstract

Alport disease in humans, which usually results in proteinuria and kidney failure, is caused by mutations to the *COL4A3*, *COL4A4*, or *COL4A5* genes, and absence of collagen α3α4α5(IV) networks found in mature kidney glomerular basement membrane (GBM). The Alport mouse harbors a deletion of the *Col4a3* gene, which also results in the lack of GBM collagen α3α4α5(IV). This animal model shares many features with human Alport patients, including the retention of collagen α1α2α1(IV) in GBMs, effacement of podocyte foot processes, gradual loss of glomerular barrier properties, and progression to renal failure. To learn more about the pathogenesis of Alport disease, we undertook a discovery proteomics approach to identify proteins that were differentially expressed in glomeruli purified from Alport and wild-type mouse kidneys. Pairs of cy3- and cy5-labeled extracts from 5-week old Alport and wild-type glomeruli, respectively, underwent 2-dimensional difference gel electrophoresis. Differentially expressed proteins were digested with trypsin and prepared for mass spectrometry, peptide ion mapping/fingerprinting, and protein identification through database searching. The intermediate filament protein, vimentin, was upregulated ∼2.5 fold in Alport glomeruli compared to wild-type. Upregulation was confirmed by quantitative real time RT-PCR of isolated Alport glomeruli (5.4 fold over wild-type), and quantitative confocal immunofluorescence microscopy localized over-expressed vimentin specifically to Alport podocytes. We next hypothesized that increases in vimentin abundance might affect the basement membrane protein receptors, integrins, and screened Alport and wild-type glomeruli for expression of integrins likely to be the main receptors for GBM type IV collagen and laminin. Quantitative immunofluorescence showed an increase in integrin α1 expression in Alport mesangial cells and an increase in integrin α3 in Alport podocytes. We conclude that overexpression of mesangial integrin α1 and podocyte vimentin and integrin α3 may be important features of glomerular Alport disease, possibly affecting cell-signaling, cell shape and cellular adhesion to the GBM.

## Introduction

The kidney glomerulus is a unique, semipermeable capillary tuft that allows the passage of plasma water and small solutes into the tubular portion of the nephron, while retaining albumin and larger molecules in the circulation. Diseases affecting the glomerular barrier properties commonly result in the loss of circulating plasma proteins into the urine, a condition called proteinuria, and unchecked proteinuria can lead to end stage renal disease requiring dialysis and/or kidney transplantation. The filtration barrier itself is comprised of the fenestrated glomerular endothelium with its glycocalyx and loosely attached cell coat [1], the glomerular basement membrane (GBM), and the visceral epithelial podocytes with their intervening slit diaphragm complexes [2]. The endothelium, GBM, and podocytes are all necessary and work synergistically in maintaining the glomerular filtration barrier. The importance of the GBM for glomerular barrier properties in humans is underscored by Alport disease. Affected individuals harbor mutations to any one of the three genes encoding the type IV collagen network found in mature GBM; *COL4A3*, *COL4A4*, or *COL4A5*, and Alport patients usually suffer a progressive loss of barrier function, splitting of the GBM, and, eventually, renal failure [3].

Depending upon the tissue location, basement membranes contain just one of three different heterotrimers of type IV collagen chains: α1α2α1(IV), α3α4α5(IV), or α5α6α5(IV). These triple helices further associate to form a three-dimensional network of polymerized collagen IV [4]. During mammalian kidney development, the type IV collagen composition of the GBM undergoes isoform substitution. Whereas GBMs of immature glomeruli contain networks of α1α2α1(IV) heterotrimers, GBMs of fully developed glomeruli contain networks of α3α4α5(IV), which is the primary type IV collagen species that persists into adulthood [5]. Laminin is another heterotrimeric glycoprotein that forms polymers in basement membranes and it also undergoes isoform substitution during glomerular development. Specifically, laminin α1β1γ1 is present in the earliest GBMs of immature glomeruli whereas fully mature GBMs contain only laminin α5β2γ1 [6].

Mechanisms accounting for GBM collagen IV and laminin isoform switching are unknown, but these substitutions appear to be required for the acquisition and maintenance of glomerular filtration barrier properties. As already mentioned, mutations to genes encoding the collagen α3α4α5(IV) heterotrimer result in Alport disease and cause structural and functional deficits to the glomerulus. Similarly, mutations to *LAMB2*, which encodes the laminin β2 chain, cause Pierson syndrome, which results in ocular defects, congenital nephrosis, and renal failure usually within a few weeks after birth [7].

Two genetically engineered mouse models of Alport disease have been produced by deletion of the *Col4a3* locus [8], [9]. Without the collagen α3(IV) chain, a stable α3α4α5(IV) heterotrimer can not form, and GBMs lack this collagen IV isoform altogether. Although disease severity differs depending upon strain [10], both of the genetic mouse models parallel key aspects of human Alport kidney disease progression. Specifically, *Col4a3* null mice are viable, and kidney function appears normal until the onset of proteinuria at ∼5 weeks of age. Like Alport patients, mouse mutants retain collagen α1α2α1(IV) in their GBMs into adulthood, and there is also ectopic expression laminins α1, α2 and β1 in peripheral loop GBM [11], [12], especially in the irregular subepithelial thickenings that are typical of Alport glomeruli [13].

Whereas the collagen α1α2α1(IV) seen in immature GBM, as well as the ectopic laminins of Alport mouse GBM, originate from both endothelial cells and podocytes, the podocyte alone is responsible for the synthesis of collagen α3α4α5(IV) found in mature GBM [14]. The progression of Alport syndrome in humans and in mouse models ultimately leads to end stage renal disease, but this is a relatively slow process compared to other podocyte mutations. For example, mutations affecting *NPHS1* (encoding the slit diaphragm protein, nephrin) or *NPHS2* (encoding the slit diaphragm-associated protein, podocin), result in renal failure and death within a few days after birth [15], [16].

Although the α1α2α1(IV) collagen retained in Alport GBMs is apparently able to compensate partially for the absence of α3α4α5(IV) collagen, the later isoform has more cysteine residues available for disulfide crosslinks between α chains, which may confer improved resistance of the GBM to hydrostatic pressure within the glomerular capillary [17]. Alport GBM has also been shown to be more susceptible to proteolysis *in vitro* [17], and mechanical strain induces metalloprotease expression in podocytes [18], which is also upregulated in Alport [19]. Glomeruli from *Col4a3*-null mice are more easily deformable early in disease progression, as measured by a combination of atomic force microscopy and capillary micromechanics [20], and they are more permeable to intravenously injected ultrastructural tracers such as ferritin [21].

The morphologic course of Alport disease in humans and in mouse models has been well described, but there is much yet to learn on how the abnormal GBMs affect mesangial cells, endothelial cells and podocytes, and cause proteinuria. In an effort to advance this question, we isolated glomeruli from Alport and wild-type mice and undertook a proteomics approach to determine which proteins were differentially expressed.

## Results

Glomeruli were isolated from kidneys of three 5 week old *Col4a3* knockout mice and three age-matched wild-type controls. Three samples were prepared consisting of equal protein concentrations of glomerular lysates from each genotype (wild-type Col4a3^+/+^ lysate labeled either with Cy3 or Cy5 and knockout Col4a3^−/−^ lysate [with opposite fluorescent tag]), and proteins in each mixture were separated by two-dimensional difference gel electrophoresis (2D DIGE). The three resulting gels were each fluorescently scanned and individual spot signals were calculated, and then averaged for the three gels. Spots with significant increases or decreases in differential intensities (p<0.05) were robotically picked for analysis by MALDI-TOF (matrix-assisted laser desorption/ionization-time of flight) peptide mass fingerprinting and TOF/TOF peptide fragmentation followed by data-base searching to produce statistically significant candidate protein matches. This resulted in the identification of 9 differentially expressed proteins with molecular weight search (MOWSE) scores of greater than 55 (95% confidence interval), and these are listed in Table 1. Notably, the proteins with largest expression differences between Alport and wild-type were both cytoskeletal: the intermediate filament (IF) protein, vimentin, was upregulated ∼2.5 fold in Alport, and the microtubule protein, β-tubulin, was downregulated ∼2.4 fold in Alport (Table 1).

**Table 1:** Proteins altered in Alport glomeruli identified by 2D DIGE and MALDI-TOF.

Gene Name	Gene symbol	Protein ID	Fold change	p-value	Molecular Mass (kDa)	pI	MOWSE score	qPCR fold	qPCR p-value
**Increased in Alport glomeruli**									
Vimentin	Vim	P20152	2.48	0.01	53.7	5.1	473	5.24	0.006
Annexin A3	Anxa3	O35639	1.68	0.001	36.5	5.3	371	2.18	0.01
**Decreased in Alport glomeruli**									
Tubulin, beta-5	Tubb5	P99024	−2.41	0.047	50	7.8	73	n/c	n/a
Dihydropyrimidinase-like 2	Dpysl2	O08553	−2.13	0.009	62.6	5.9	85	n/c	n/a
Beta actin	Actb	P60710	−1.98	0.015	41.7	5.3	109	n/c	n/a
Glutamyl aminopeptidase	Enpep	P16406	−1.87	0.042	108.4	5.3	108	n/c	n/a
Collagen type VI, alpha 1	Col6a1	Q04857	−1.82	0.003	109.5	5.2	70	n/c	n/a
DEAD (Asp-Glu-Ala-Asp) box polypeptide 39B	Ddx39b	Q9Z1N5	−1.54	0.024	49.5	5.4	69	n/c	n/a
Prohibitin	Phb	P67778	−1.35	0.016	29.8	5.5	107	n/c	n/a

n/c = no change, n/a = not applicable.

To determine whether mRNAs were altered in Alport, primers were designed to mRNA of the 9 differentially expressed proteins and total glomerular RNA was isolated from 4 week old wild-type and Col4a3^−/−^ Alport mice. Quantitative real time RT-PCR (qPCR) showed that glomerular mRNA signals were significantly increased for vimentin (upregulated 5.24 fold, p<0.006) and the calcium-dependent, phospholipid binding protein, annexin A3 (upregulated 2.18 fold, p<0.01) in Alport (Table 1). No statistically significant changes in mRNAs were found for any of the proteins shown to be decreased in Alport glomeruli (Table 1).

**Figure 1:**
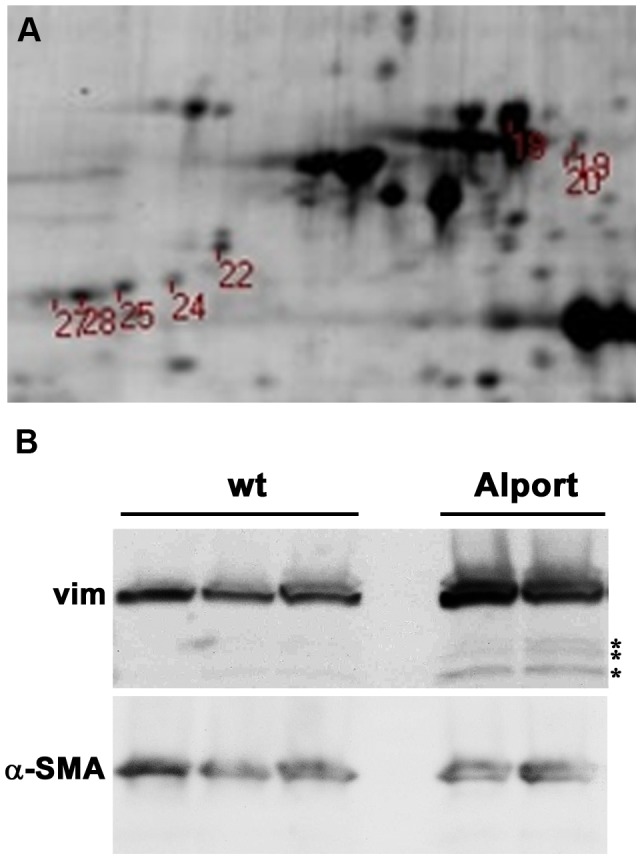
The intermediate filament protein vimentin is upregulated in Alport glomeruli. A: A digital scan of a portion of the 2D gel showing the position of the 8 vimentin spots robotically picked for LC-MS/MS. B: Western blot of wild-type (wt) or Alport mouse glomerular lysates harvested at 4 weeks of age probed with goat anti-vimentin IgGs (Vim, upper blot), then stripped and re-probed with mouse anti-smooth muscle actin (α-SMA, lower blot) as a loading control. Asterisks (*) indicate lower molecular weight bands that are more prominent in the Alport glomerular lystates, possibly representing proteolytic fragments of vimentin.

We chose to focus on vimentin, as expression of this IF protein has been shown previously to be restricted in glomeruli to podocytes [22]–[24]. Further, this is the glomerular cell type that synthesizes the collagen α3α4α5(IV) heterotrimer found in mature GBM [14], which is lacking in Alport. Indeed, 8 different resolved protein spots (migrating at different positions) identified by DIGE were verified as vimentin (Fig. 1A). These different forms of vimentin may have represented degradation products, or perhaps species of vimentin with different post-translational modifications that altered their charge. Western blots of isolated glomerular lysates from wild-type (n = 3) or Alport (n = 2) kidneys showed that vimentin migrated as a major ∼50 kD band, with an obvious, increased abundance in Alport glomeruli (Fig. 1B). Blots showed some minor, lower molecular weight bands reacting with anti-vimentin antibodies that were also more prominent in Alport samples (asterisks, Fig. 1B) than those from wild-type mice, and these may also have represented proteolytic vimentin fragments. Blots were stripped and re-probed with anti-α-smooth muscle actin, which served as a loading control (α-SMA, Fig. 1B).

**Figure 2:**
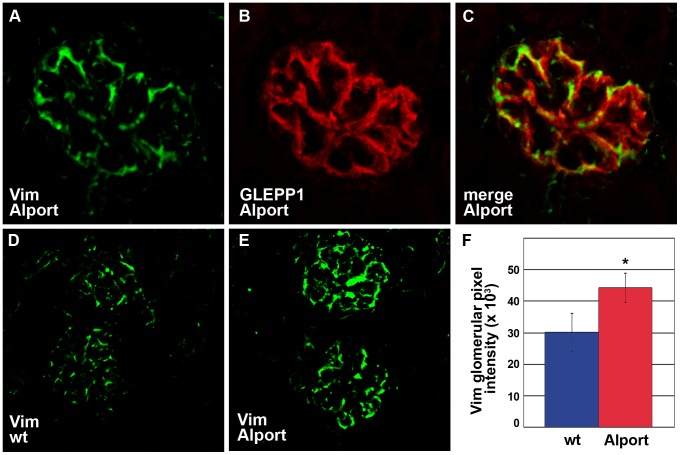
Vimentin is upregulated in podocytes of Alport glomeruli. A–C: Fresh frozen kidney sections from Alport mice were labeled with a combination of goat anti-vimentin and rabbit anti-GLEPP1 IgGs, followed by the appropriate species-specific Alexa Fluor secondaries. Vimentin labeling (A) is restricted to the epithelial podocyte layer, marked by GLEPP1 staining (B), overlap of staining is shown in C (merge). D–F: Representative fluorescence micrographs are shown of anti-vimentin labeling (Vim) of wild-type (D, wt), or Alport (E) mouse glomeruli. The relative glomerular fluorescence intensities were measured and averaged for n = 3 mice of each genotype, wildtype (wt, blue) or Alport (red). * p = 0.04.

To confirm expression of vimentin in Alport mouse glomeruli, frozen kidney sections from Alport mice were immunolabeled with anti-vimentin, and antibody appeared to be bound specifically to podocytes (Fig. 2A). This was verified using double immunolabeling with podocyte-specific, anti-GLEPP1 IgG (Fig. 2B) [25], and merged images showed considerable immunofluorescence overlap (Fig. 2C). To certify the upregulation of vimentin in Alport glomeruli, the immunofluorescence signals of bound anti-vimentin antibody to glomeruli of wild-type (Fig. 2D) and Alport mice (Fig. 2E) were quantified [21]. Glomerular expression of vimentin was significantly increased in Alport (Fig. 2F, 1 tail t-test, p<0.05), but the expression of GLEPP1 did not change in these samples (not shown).

**Figure 3:**
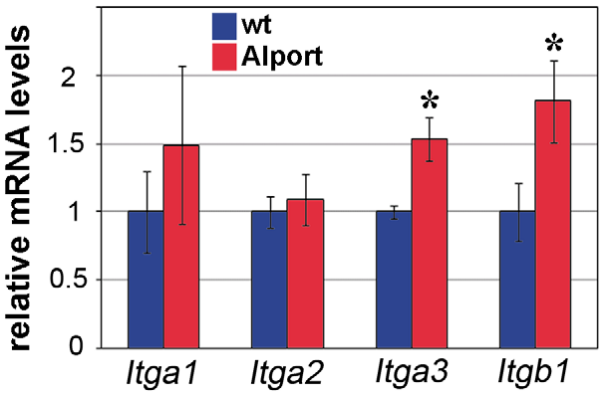
The mRNA levels encoding *Itga3* and *Itgb1* are upregulated in Alport glomeruli. Quantitative real time RT-PCR was performed on n = 3 wild-type (wt, blue) and n = 3 Alport (red) glomerular RNA isolated at 4 weeks of age. Both *Itga3* and *Itgb1* mRNAs are significantly increased in Alport glomerular RNA. * p = 0.02.

We next assessed how the absence of collagen α3α4α5(IV) in the GBM might have affected the composition of the internal IF cytoskeleton of the Alport podocyte, reasoning that the matrix receptors, integrins, may have been involved. Integrins have been implicated in the Alport mouse model previously [11], but a comprehensive study of their expression in Alport has not been undertaken. Knowing that the collagen IV and laminin composition of the GBM are both abnormal in Alport disease, we selected a subset of integrins for analysis that likely represented the most prominent collagen IV and laminin receptors. Quantitative real time RT-PCR showed statistically significant increases in mRNAs encoding integrin α3 and integrin β1 in Alport glomeruli, but no significant changes were detected for integrin α1 or integrin α2 mRNAs (Fig. 3).

**Figure 4:**
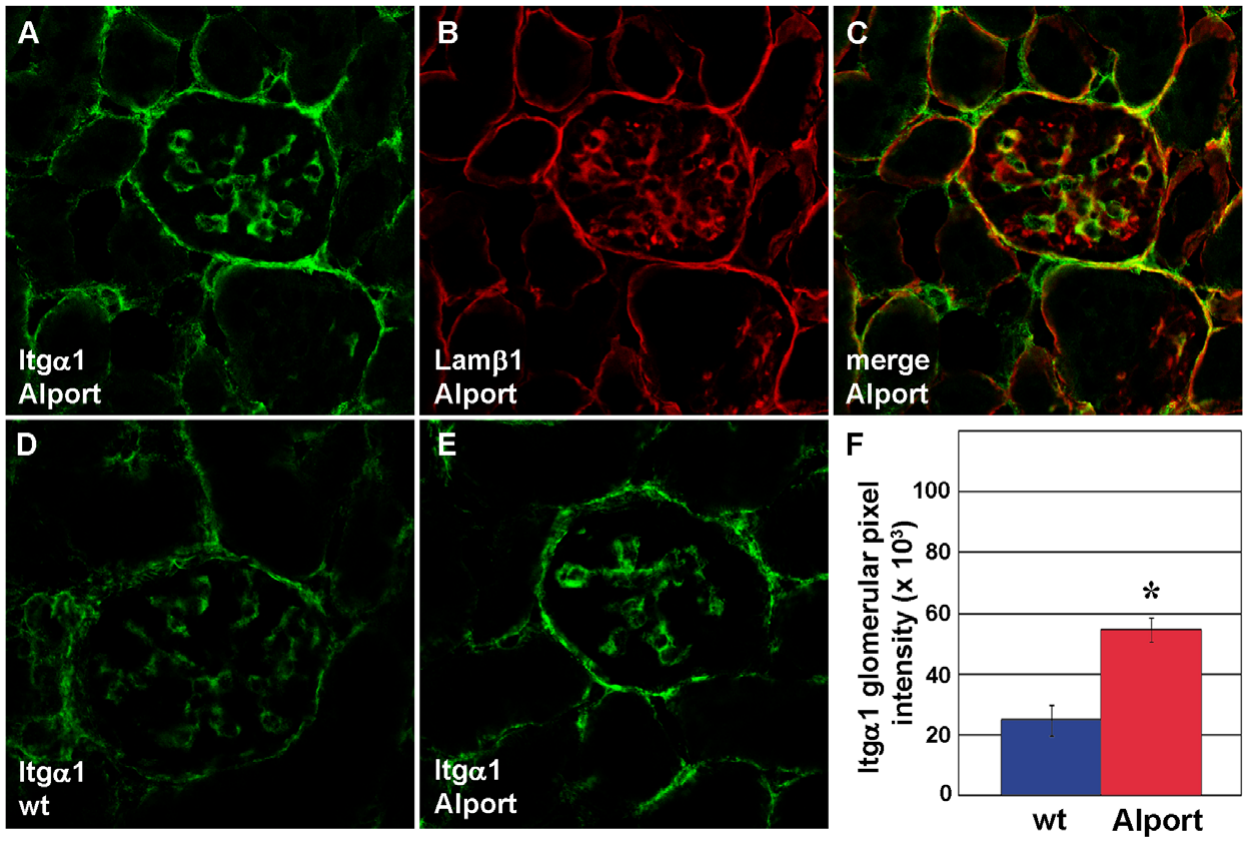
Integrin α1 protein is upregulated in the mesangium of Alport glomeruli. A–C: Fresh frozen kidney sections from 4 week old Alport mice were labeled with a combination of hamster anti-integrin α1 and rat anti-laminin β1 IgGs, followed by the appropriate species-specific Alexa Fluor secondary antibody. Anti-integrin α1 labeling (A, Itgα1) is restricted to the mesangial layer, marked by anti-laminin β1 staining (B, Lamβ1), and overlap of staining is shown in C (merge). D–F: Representative fluorescence micrographs are shown of anti-integrin α1 labeling of wild-type (D, wt), or Alport (E) mouse glomeruli. Glomerular fluorescence intensities were averaged for n = 3 mice of each genotype, wildtype (wt, blue) or Alport (red), and integrin α1 signals were significantly greater in Alport. * p = 0.01.

**Figure 5:**
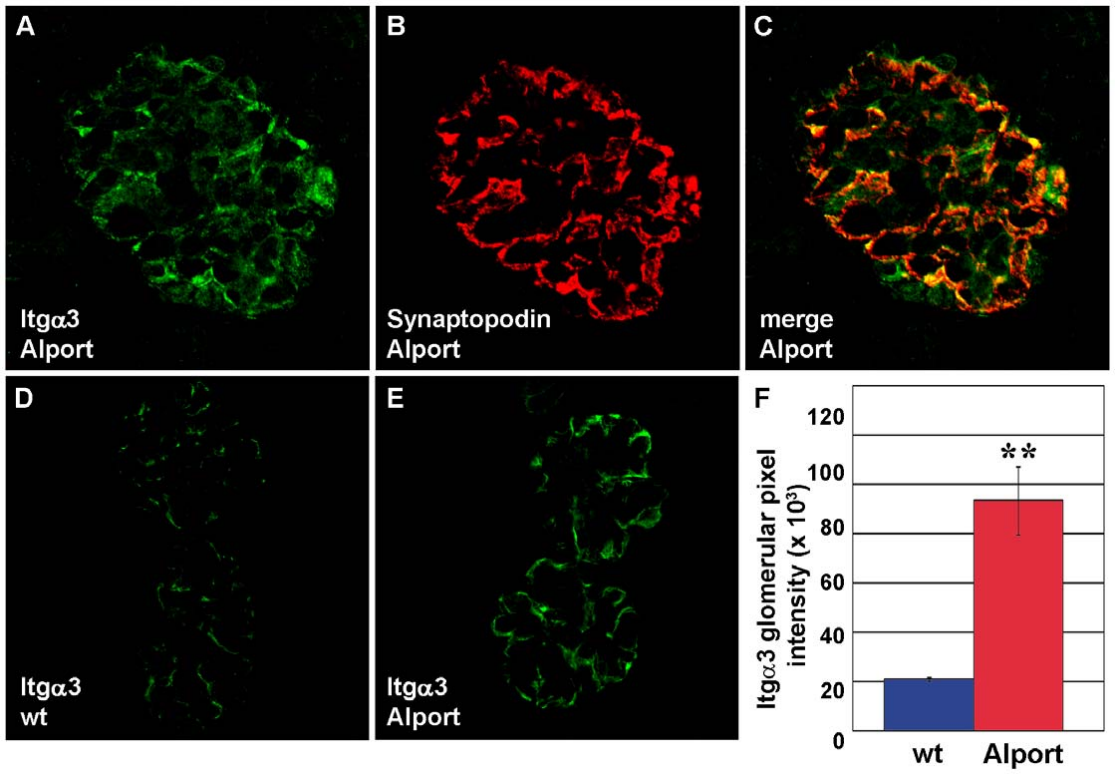
Integrin α3 protein is upregulated in podocytes of Alport glomeruli. A–C: Fresh frozen kidney sections from 4 week old Alport mice were labeled with a combination of rabbit anti-integrin α3 and mouse anti-synaptopodin IgGs, followed by the appropriate species-specific Alexa Fluor secondaries. Anti-integrin α3 immunolabeling (A) is restricted to the epithelial podocyte layer, marked by synaptopodin staining (B), and overlap of staining is shown in C (merge). D–F: Representative fluorescence micrographs are shown of anti-integrin α3 labeling of wild-type (D, wt), or Alport (E) mouse glomeruli. The glomerular fluorescence intensities were averaged for n = 3 mice of each genotype, wild-type (wt, blue) or Alport (red), and integrin α3 signals were significantly greater in Alport. * p = 0.006.

**Figure 6:**
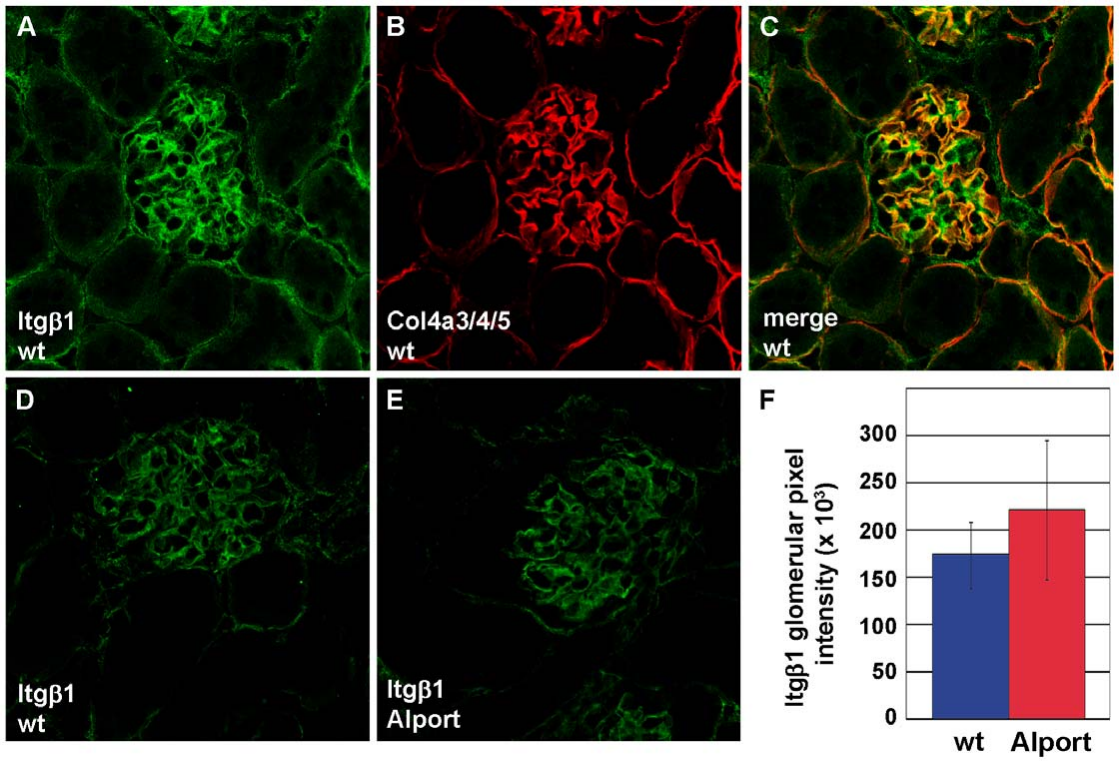
There is no change in expression of integrin β1 in Alport glomeruli. A–C: Fresh frozen kidney sections from 4 week old wild-type (wt) mice, immunolabeled with anti-integrin β1 (A), anti collagen α3α4α5(IV) (B) and overlap of labeling is shown in C (merge). D–F: Representative fluorescence micrographs of wildtype (D) and Alport glomeruli immunolabled with anti-integrin β1. Glomerular fluorescence intensities were averaged for n = 3 mice of each genotype, wild-type (wt, blue) or Alport (red), and there is no statistical difference.

We also examined and quantified the distribution of integrin receptor proteins in wild-type and Alport mouse glomeruli using confocal immunofluorescence microscopy. Integrin α1 immunolocalized to wild-type and Alport glomeruli in what appeared to be a mesangial pattern (Figs. 4A). When sections were doubly immunolabeled with anti-laminin β1 (Fig. 4B), a marker for mesangial matrix in mature glomeruli [6], there were some areas of overlap with integrin α1 (Fig. 4C). However, there were also some areas of discrete anti-integrin α1 binding as well, suggesting that some integrin α1 expression may have occurred in glomerular capillary loops (Fig. 4C). Regardless, when total integrin α1 immunolabeling intensities were quantified in wild-type (Fig. 4D) and Alport glomeruli (Fig. 4E), they were significantly higher in Alport (Fig. 4F). In contrast to the somewhat ambiguous localization of integrin α1, integrin α3 immunolocalized specifically to podocytes, as shown by co-localization with the podocyte marker, synaptopodin (Fig. 5A–C) [26]. Like integrin α1, the integrin α3 immunolabel signal intensities were also significantly increased in Alport glomeruli (Fig. 5D–F). In contrast, signal intensities for integrin β1, which localized to GBM loops and mesangial matrices (Fig. 6), were no different in Alport when compared to wild-type (Fig. 6).

## Discussion

Our study began with a discovery proteomics approach applied to glomerular lysates isolated from 5 week old Alport and wild-type mouse kidneys and these results were validated by multiple secondary studies. The DIGE-MS approach revealed changes in a relatively small number of proteins, which is not particularly surprising, given that many proteins in the glomerular extracellular matrix are difficult to solubilize under conditions compatible with 2D gel electrophoresis. Additionally, larger macromolecular protein assemblies would probably not be captured by this analysis if they were not fully denatured. Multiple forms of the protein, vimentin, which comprises a class of IFs commonly found in mesenchymal cells, had the largest magnitude increase in Alport. Upregulation of vimentin gene transcription was confirmed by qPCR of mRNA harvested from isolated Alport glomeruli, and confocal microscopy of kidney sections immunolocalized overexpressed vimentin protein specifically to Alport podocytes. Reasoning that signals resulting in podocyte IF reorganization might have been transmitted through the basement membrane protein receptor integrins, we evaluated integrin mRNA and protein expression in Alport glomeruli. Compared to wild-type, there were significant increases in *Itga3* and *Itgb1* mRNAs in Alport, and integrin α1 and α3 protein immunofluorescence signals were increased as well. We speculate that the Alport GBM lacking collagen α3α4α5(IV) caused changes in integrin expression/distribution, which directly and/or indirectly altered the organization of the podocyte IF cytoskeleton, affecting cell shape and possibly signal transduction and gene transcription. Alternatively, increases in vimentin availability and/or distribution within the podocyte may have affected podocyte expression and/or turnover of integrins, as discussed further below. Dysregulation of integrins and podocyte IFs may therefore be key pathogenic components of glomerular Alport disease.

IFs are thin, ∼10 nm diameter, intracellular filaments and are one of three interconnected cytoskeletal systems found within cells (the other two being actin microfilaments and tubulin-based microtubules) [27]. Depending upon the cell type, IFs are composed of as many as ∼70 evolutionarily related proteins including acidic and neutral based keratins (epithelial cells), desmin (muscle), vimentin (mesenchymal cells), and neurofilaments (neurons), although heteropolymers can exist as well. Originally believed to be a static network that provided cells a method to resist deformation and mechanical stress, IFs are now known to be highly dynamic, motile elements, that extend from the cytoplasm into the nucleoplasm. Cytoplasmic intermediate filament proteins exist as aggregates or particles, short filaments called “squiggles,” and long fibrils [27], [28]. All have the capacity to shorten and elongate bidirectionally through their association with molecular motors such as kinesin and dynein. In general, cytoplasmic IFs provide biomechanical integrity to cells but they also participate in cell signaling cascades, help regulate delivery and compartmentalization of stress-activated kinases, and they are active in cell-matrix adhesion and directional migration [27], [28]. The intranuclear network of IFs are composed of polymers of lamin A, B1 and B2, C and other lamin isoforms. The IF nucleoskeleton provides a structural framework that facilitates DNA repair, replication, transcription and modulates the architecture of chromatin [27]–[29].

Among the many different IF proteins, vimentin is one of the best characterized [30]. Like other IF family members, vimentin contains a central, α helical rod domain and variable, non-α helical N-terminal head and C-terminal tail domains that contain several phosphorylation sites. Vimentin monomers associate in parallel to form a coiled-coil dimer, and the degree of assembly/disassembly of vimentin into filament polymers is regulated by the dephosphorylation/phosphorylation status of the vimentin head and tail domains [31]. In other words, dephosphorylation induces vimentin IF assembly, and phosphorylation induces disassembly. There are a number of other post-translational modifications of vimentin as well, including citrullination, sumoylation, and *O*-GlcNac derivatization, all of which can affect vimentin structure and function [32].

Attempts to define specific physiologic functions for vimentin through gene targeting were originally inconclusive, as vimentin knockout mice did not demonstrate an overt phenotype [33]. Later, however, vimentin knockouts were shown to have glial abnormalities causing cerebellar and motor coordination deficits [34], and impaired wound healing reflecting delayed fibroblast migration and decreased wound contraction [35]. Other reports showed that vimentin knockouts display reductions in lymphocyte binding to endothelial cells and less vascular transmigration due to decreases in expression of integrin β1 on lymphocytes and ICAM-1 and VCAM-1 on endothelial cells [36].

The relatively abundant presence of vimentin in mammalian podocytes has been known for some time [23], [37], [38]. Vimentin appears to be associated with another IF protein, nestin, in the cell body and primary processes of podocytes, and some studies show an extension of vimentin into the actin microfilament- and microtubule-rich terminal foot processes [24]. Upregulation of vimentin and reorganization of podocyte IFs have also previously been observed in rats with puromycin aminonucleoside nephrosis [39], [40], mice with podocyte-selective deletion of the microRNA generating enzyme, dicer, which results in podocyte foot process effacement, split GBMs, and proteinuria [41], and in human glomerular diseases [42], [43]. The overexpression of vimentin seen here and in the examples cited above is probably related to podocyte shape change that leads to broadening of foot processes during effacement, but may reflect other intracellular activities of vimentin in response to podocyte injury. Among other functions, vimentin is now known as a key regulator of cell adhesion through its direct and indirect interaction with integrins [30].

Integrins mediate cell-cell and cell-extracellular matrix (ECM) interactions and are comprised of non-covalent heterodimers of transmembrane protein α and β subunits [44]. Their large N-terminal ectodomains bind extracellular ligands (such as basement membrane laminins and type IV collagen), and their short C-terminal cytoplasmic tails bind cytoskeletal elements and signal transduction proteins [44]. Depending upon the cell type, the integrin isoform(s) it expresses, and available substrates, integrins can become organized into specific cell adhesion structures. For example, in keratinocytes, the ectodomain of integrin α6β4 binds to laminin while the cytoplasmic domain of integrin β4 binds to plectin, which loosely connects the integrin to intracellular keratin IFs [30]. This association is strengthened by recruitment of bullous pemphigoid protein 180 (BP180), which then links to keratin IFs through another protein, BP230. Together, this complex forms a hemidesmosome, which is a highly stable cell-ECM adhesion junction that anchors keratinocytes firmly to the dermal-epidermal junctional basement membrane [30]. Hemidesmosomes are recognized ultrastructurally by the presence of a discrete electron dense plaque just internal to the basal plasma membrane, and an accumulation of IFs that radiate apically from the plaque. Notably, hemidesmosomes are absent in the basal membranes of glomerular endothelial cells and podocytes, as well as in mesangial cell membranes.

Vimentin-associated matrix adhesions (VAMs) are similar to hemidesmosomes in that integrin αvβ3 binds to vimentin IFs through plectin [30], although in endothelial cells, integrin α2β1 associates with vimentin directly [45]. Unlike desmosomes, there is no intracellular electron dense plaque in VAMs, and actin microfilaments can also be present. Also unlike desmosomes, VAMs are dynamic, transient structures important for cell migration and shape change [30].

In addition to its involvement with integrin-dependent cell-matrix adhesion, vimentin also mediates the vesicular trafficking of integrins to the plasma membrane during integrin turnover [30]. Specifically, unphosphorylated vimentin oligomers have been shown to bind to endocytic vesicles bearing integrins. Phosphorylation of vimentin N-terminal serines by PKCε decouples vimentin from vesicle membranes, which allows the vesicles bearing integrins to recycle to and fuse with the plasma membrane [30], [46]. The uncoupled phospho-vimentin-PKCε complex then associates with vimentin polymers that are dephosphorylated, PKCε dissociates, vimentin binds to incoming vesicles, and the cycle repeats [30], [46]. Inhibition of vimentin phosphorylation results in accumulation of integrins within intracellular vesicles, reduction of integrins at the plasma membrane, and loss of directional cell motility [30], [46].

Although integrin proteins were not detected in the discovery proteomics phase of this study, the findings for vimentin implicated a role for integrins, and we therefore tested this new hypothesis with additional experimentation. Specifically, there were significant increases in mRNAs encoding integrin α3 and β1 from isolated Alport glomeruli, more integrin α1 protein in Alport mesangial cells, and more integrin α3 protein in Alport podocytes. Integrin α1β1, believed to function primarily as receptor for type IV collagen [47], has been shown previously to be upregulated in proliferating mesangial cells in glomerulonephritis [48], [49]. This integrin also negatively mediates collagen IV synthesis and integrin α1 null mice suffer more severe glomerular fibrosis after renal injury [50], [51]. On the other hand, Alport mice with genetic deletion of integrin α1 experience less mesangial matrix expansion and reduced podocyte foot process effacement [11]. Clearly, additional work is needed to determine what role upregulated integrin α1 plays, if any, in Alport mesangial cells.

Integrin α3β1, believed to function primarily as a receptor for laminins [52], has been shown to be critical for development and maintenance of glomerular capillary loops. Global integrin α3 knockouts die at birth with severe glomerular abnormalities including disorganized GBMs and podocyte effacement [53], and the same result is obtained in podocyte-specific, conditional mutants [54]. Laminin overexpression in the GBM has been observed previously in human Alport patients and in dog and mouse models of Alport disease [12], [13], and perhaps the upregulation of integrin α3 in Alport podocytes seen here reflects the increased presence of its laminin ligand in the diseased GBM. Alternatively, the overexpression of vimentin within podocytes may have influenced integrin trafficking to their basal membranes, possibly affecting podocyte adherence and glomerular barrier properties.

In summary, our results show that an absence of collagen α3α4α5(IV) in the Alport GBM resulted in increased expression of mesangial cell integrin α1 and podocyte integrin α3 and vimentin. Much future work is necessary before we can learn how the Alport GBM induced changes in patterns of integrin and vimentin expression, whether these changes were linked directly, indirectly, or independent from one another, and how they might have contributed to the progression of Alport glomerulopathy. Nevertheless, these findings suggest that an altered distribution of glomerular integrins and vimentin are likely to be important for the pathogenesis of Alport disease.

## Materials and Methods

### Animals

#### Ethics Statement

All experiments with mice strictly followed policies and procedures established by the Animal Welfare Act and the Public Health Service Policy on the Humane Care and Use of Laboratory Animals. The experimental protocol was approved by the Institutional Animal Care and Use Committee at the University of Kansas Medical Center (protocol number 2011-1972). Surgeries were conducted while mice were deeply anesthetized with ketamine HCl-xylazine, and all efforts were taken to minimize suffering.

Mice with a targeted deletion encoding the non-collagenous one (NC1) domain of the *Col4a3* gene on the 129/SvJ background have been described previously [8], and were obtained from the Jackson Laboratory (129-Col4a3^tm1Dec/J^, Bar Harbor, ME). Mice were genotyped using the polymerase chain reaction (PCR).

### Glomerular isolation

Glomeruli were collected from 5 week old *Col4a3* null and wildtype littermate controls (n = 3 of each genotype) by the magnetic bead perfusion technique [55]. Briefly, mice were anesthetized with 1 mg/10 grams body weight ketamine and 0.15 mg/10 grams body weight xylazine. Blood was washed from the animals by perfusion of the heart with Hank's balanced salt solution (HBSS) followed by intracardiac injection of 2×10^6^ Dynabeads M-450/ml (Invitrogen, Carlsbad, CA) in HBSS. After 40 ml was perfused, kidneys were removed and minced with a razor blade on ice, followed by digestion at 37°C with 1 mg/mL collagenase and 100 U/ml DNase I for 30 minutes. Digested kidneys were filtered twice with 100 micron Falcon cell strainers, and tissue was pelleted by gentle centrifugation (200 g, 5 minutes). Glomeruli were isolated with a DynaMag-2 magnetic particle concentrator (Invitrogen), and resuspended in HBSS containing protease inhibitors (1∶100 of protease inhibitor cocktail (PIC), (Sigma-Aldrich Co., St. Louis, MO), and 0.1 mM phenylmethylsulfonyl fluoride, PMSF). After two additional rounds of magnetic isolation and resuspension, the glomeruli were snap frozen in liquid nitrogen and stored at −80°C.

### 2D gel and mass spectrometry analysis

Glomerular proteins were solubilized with extraction buffer (7 M urea, 2 M thiourea, 4% CHAPS, 25 mM DTT, 5 mM EDTA, and 30 mM Tris-HCl, pH 8.0) for 20 minutes at room temperature. Magnetic beads and insoluble debris were removed by centrifugation and soluble proteins were recovered in the supernatant. Protein concentration was determined with a 2-D Quant kit (GE Healthcare, Piscataway, NJ), and adjusted to 2.0 mg/ml.

The mixed internal standard methodology was used as previously described [56], with the following modifications. Briefly, individual glomerular extracts from wild-type or Alport mice were labeled with cy3 or cy5 such that two of the three samples from a given group were labeled with the same dye (such as cy3) and the third sample with the other dye (cy5) to avoid any dye-labeling bias in the data. Cy3/5 pairs of wild-type/Alport samples were then mixed with an aliquot of a cy2-labeled mixture of all six samples, which served as an internal standard. The resulting tripartite mixtures were then added to unlabeled glomerular protein from wild-type animals, and 0.5 mg total protein was loaded per 2D gel. All 2D DIGE equipment was manufactured by GE Healthcare. The resulting three sets of glomerular protein mixtures were then resolved by isoelectric focusing (24 cm IPG pH 4–7) using a manifold-equipped IPGphor II, followed by 12% SDS-PAGE (to separate proteins ranging from ∼12–150 kDa) in a DALT12 electrophoresis chamber using hand-cast gel cassettes with one plate treated with bind-silane to facilitate robot spot excision, all using the manufacturer's recommended protocols. Fluorescent images consisting of 16-bit .tiff files were acquired at 100 micron resolution at each mutually exclusive excitation/emission setting for cy2, cy3, or cy5 using a Typhoon Multivariable Imager, per the manufacturer's protocol.

DIGE expression values and univariate statistical analysis was carried out using DeCyder-2D v6.5 (GE Healthcare), which normalized the ratios across all six samples relative to the cy2 signal on each gel for each specific protein, one-by-one, thereby reducing influence due to gel-to-gel variation. The threshold for significant change in relative protein abundance was set at >1.5 fold, which was greater than 2 standard deviations of the mean abundance change when considering only pair-wise, wild-type/Alport comparisons on each gel separately.

DIGE gels were post-stained with Spyro Ruby (Molecular Probes, Carlsbad, CA) and proteins of interest were excised directly from these gels and processed for in-gel trypsin protease digestion and preparation for mass spectrometry using an automated Ettan Spot Handling Workstation (GE Healthcare). Peptide ion mapping/fingerprinting was carried out on a Voyager 4700 mass spectrometer (Applied Biosystems/Life Technologies, Carlsbad, CA); MALDI-MS was internally calibrated using trypsin autolytic fragments in digests to provide mass accuracy to within 20 ppm. Additional TOF/TOF tandem mass spectrometry provided fragmentation data on selected peptide ions which was used in conjunction with the peptide ion masses in database searches to provide statistically significant candidate protein identifications. The Mascot database search algorithm (Matrix-Science, Boston, MA) was used for protein identification, searching against the complete Swiss-Prot and NCBInr databases (2009 database release dates) without constraining protein molecular weight, isoelectric point, or molecular species. Carbamidomethylation of cysteines was performed and partial oxidation of methionine residues was allowed in search parameters. Species constraints were invoked for second-pass searches as needed. Additional mass spectrometry using liquid chromatography coupled with tandem mass spectrometry was carried out for a few proteins of interest using an LTQ linear ion trap mass spectrometer (Therm Fisher Scientific, Waltham, MA), coupled with a nanoflow HPLC pump (Eksigent, Framingham, MA) running at 5 µl/minute. The peptides were resolved on a hand made fused silica capillary column, 100 µm I.D. ×15 cm, pressure packed with C18 resin (Jupiter C_18_, 5 µm, 300 Å, Phenomonex, Torrance, CA). The resulting spectra were searched against the mouse database using the Sequest algorithm [57], with data filtering and analysis using IDPicker [58].

### qPCR

RNA was extracted from glomerular samples (n = 3 of each genotype) using RNeasy micro kits (Qiagen, Valencia, CA), quantified and diluted to 10 ng/µl. Real time PCR was carried out using gene-specific primers (Table 2) and a QuantiTect SYBR Green RT-PCR kit (Qiagen) using an iCycler (Bio-Rad, Hercules, CA). The primer sets were validated for efficiency by the comparative Ct method [59], using standard curve analysis. RT-PCR products were sequenced and verified with the Basic Local Alignment Search Tool (BLAST, http://www.ncbi.nlm.nih.gov/BLAST) and/or analyzed as single peaks on melt curve analysis.

**Table 2:** Primers used in this study.

Gene Symbol Accession	Primer designation	Primer sequence	Product length (bp)
Actb NM_007393.3	ActbP64For	5′-CTAAGGCCAACCGTGAAAAG-3′	104
	ActbP64Rev	5′-ACCAGAGGCATACAGGGACA-3′	
Anxa3 NM_013470.2	Anxa3P27For	5′-TCTATACTCGGCCATCCAATC-3′	131
	Anxa3P27Rev	5′-GATTTTGAGGCGGTCCAC-3′	
Col6a1 NM_009933.3	Col6a1P97For	5′-GACATCCAGGGCTCCAAA-3′	70
	Col6a1P9Rev	5′-AGGTGTCGAGCACGAAGAAT-3′	
Ddx39b NM_019693.3	Bat1aP106For	5′-CCGGCTTCCGAGATTTTC-3′	96
	Bat1aP106Rev	5′-GGATGCACTCATGCTGGAC-3′	
Dpysl2 NM_009955.3	Dpysl2P68For	5′-AAGCCCTTCCCTGACTTTGT-3′	72
	Dpysl2P68Rev	5′-AGGGACCCCTCTCAGCTC-3′	
Enpep NM_007934.3	EnpepP83For	5′-TGGACTCCAAAGCTGATCCT-3′	76
	EnpepP83Rev	TCAGCCCATCTGACTGGAAT-3′	
Itga1 NM_001033228.1	Itga1P18For	5′- TATCCTCCTGAGCGCCTTT -3′	85
	Itga1P18Rev	5′- TGGCCTTTTGAAGAATCCAA -3′	
Itga2 NM_008396.2	Itga2P5For	5′-GGTTCTGCAGGATAGAAACCA-3′	75
	Itga2P5Rev	5′-TGGACACCGTCTTCAGTAGAAA-3′	
Itga3 NM_013565.2	Itga3P17For	5′- TCAACATGGAGAACAAGACCA -3′	90
	Itga3P17Rev	5′- CCAACCACAGCTCAATCTCA -3′	
Itga6 NM_008397.2	Itga6P18For	5′-TCAGTATTCAGGAGTAGCTTGGTG-3′	111
	Itga6P18Rev	5′-TTTCTCTTGAAGAAGCCACACTT-3′	
Itgb1 NM_010578.1	Itgb1P19For	5′- ATGCAGGTTGCGGTTTGT -3′	73
	Itgb1P19Rev	5′- CATCCGTGGAAAACACCAG -3′	
Phb NM_008831.3	PhbP46For	5′-TGGAGGTCAGAGTGAAAGCAG-3′	86
	PhbP46Rev	5′-CCGAACTTTCCAATGGACTC-3′	
Ppia NM_008907	Cyclophilin For	5′-CAGACGCCACTGTCGCTTT-3′	132
	Cyclophilin Rev	5′-TGTCTTTGGAACTTTGTCTGCAA-3′	
Tubb5 NM_011655.5	Tubb5P16Rev	5′-CTGAGTACCAGCAGTACCAGGAT-3′	86
	Tubb5P16Rev	5′-CTCTCTGCCTTAGGCCTCCT-3′	
Vim NM_011701.3	VimP109For	5′-CCAACCTTTTCTTCCCTGAA-3′	70
	VimP109Rev	5′-TTGAGTGGGTGTCAACCAGA-3′	

### Western blots

Glomerular isolates from five week old Alport and wildtype mice were homogenized on ice in H buffer (40 mM Tris pH 7.5, 15 mM NaCl, 2 mM CaCl2), supplemented with protease inhibitors (PIC 1∶100, 0.1 mM PMSF). Samples were sheared three times by passage through a syringe fitted with a 27.5 gauge needle, and incubated in H buffer for 15 minutes on a rocker at 4°C. Samples were spun for 20 minutes at 4°C at 14,000 g following 2 freeze/thaw cycles. The pellet was sonicated in H&E buffer (40 mM Tris pH 7.5, 15 mM NaCl, 2 mM CaCl2, 10 mM EDTA, 2 mM EGTA) supplemented with protease inhibitors (PIC 1∶100, 0.1 mM PMSF). The pellet was boiled for 5 minutes in SDS sample buffer with DTT and electrophoresed on a 4–15% polyacrylamide Tris-HCl precast Bio-Rad gel. Proteins were transferred to a polyvinylidene difluoride membrane. For detection of vimentin, the membrane was incubated with a 1∶100 dilution of goat anti-vimentin (MP Biomedicals (formerly ICN), Solon, OH). The membrane was incubated for 5 minutes at 50°C in stripping buffer (100 mM beta-mercaptoethanol, 2% sodium dodecyl sulfate (SDS), 62.5 mM Tris-HCL, pH 6.7). Equal loading was confirmed by probing for mouse smooth muscle actin using a SNAP ID protein detection system (Millipore, Billerica, MA) according to the manufacturer's instructions. Bound secondary antibodies were detected using chemiluminescence. Sources for all of the antibodies used in this study are shown in Table 3.

**Table 3:** Antibodies used in this study.

Antigen	Species	Clone name	Source	Catalog # or reference	Secondary, Source
Collagen α3α4α5(IV)	Mouse	26-20	Borza, D.B.	[60]	Goat anti-mouse IgG1 AlexaFluor 594, Invitrogen
GLEPP1	Rabbit		Wiggins, R.C.	[25]	Chicken anti-Rabbit IgG AlexaFluor 594, Invitrogen
Itgα1	Hamster	Ha31/8	BD Pharmingen	550568	Goat anti-Hamster IgG AlexaFluor 488, Invitrogen
Itgα2	Rabbit	H-293	Santa Cruz	sc-9089	Chicken anti-Rabbit IgG AlexaFluor 488, Invitrogen
Itgα3	Rabbit	H-43	Santa Cruz	sc-28665	Chicken anti-Rabbit IgG AlexaFluor 488, Invitrogen
Itgα1	Hamster	HMB1-1	Santa Cruz	sc-19656	Goat anti-Hamster IgG AlexaFluor 488, Invitrogen
Lamβ1	Rat	5A2	Abrahamson, D.R.	[61]	Donkey anti-Rat IgG AlexaFluor 594, Invitrogen
Smooth muscle actin	Mouse	IA4	Sigma	A2547	Sheep anti-Mouse IgG-HRP, GE Healthcare (Western blot)
Synaptopodin	Mouse	G1D4	Biodesign	Q44590M	Goat anti-mouse IgG1AlexaFluor 594, Invitrogen
Vim	Goat		ICN (now MP Biomedicals)	64-740	Donkey anti-Goat IgG Alexa Fluor 488, Invitrogen and Rabbit anti-Goat IgG-HRP, Sigma (Western blot)

### Immunofluorescence and confocal image analysis

Kidneys were harvested from deeply anesthetized 18 or 28 day old *Col4a3* knockout or wildtype mice (n = 3 of each genotype) and were frozen immediately in isopentane chilled in a dry ice-acetone bath surrounded by Tissue Tek O.C.T. Compound (Electron Microscopy Sciences, Fort Washington, PA). For some studies, kidneys were immersed in 0.2% paraformaldehyde overnight at 4°C, rinsed in PBS, and placed in 30% sucrose before freezing. Five µm-thick frozen sections were cut on a cryostat, rehydrated and post-fixed in either cold methanol or 0.2% paraformaldehyde in phosphate-buffered saline (PBS) for 10 minutes, followed by 3, 5 minute washes in PBS. Sections were incubated in primary antibodies (Table 3) for 1 hour at room temperature. Following 3, 5 minute washes in PBS, secondary antibodies were incubated for 1 hour at room temperature. After 3, 5 minute washes, slides were mounted in Prolong-Gold antifade reagent (Molecular Probes/Invitrogen, Life Technologies, Grand Island, NY). Sections were viewed by standard epifluorescence on a Leica SM5000B microscope (Bannockburn, IL). For confocal microscopy and fluorescence intensity quantification, labeling of slides for all 6 mice (n = 3 wildtype and n = 3 *Col4a3^−/−^*) was carried out on the same day and under identical conditions. Confocal image parameters were also identical and the optical section thickness was set at 0.2 µm. Ten individual glomerular images were captured per animal with a Zeiss LSM 510 scanning laser confocal microscope (Thornwood, NY). To measure glomerular fluorescence intensities, confocal images were converted to grayscale, a digital annulus was positioned over glomeruli, and pixel intensities were counted using Image J software [62], as previously described [21].

## References

[1] Haraldsson B, Nystrom J (2012) The glomerular endothelium: New insights on function and structure. Curr Opin Nephrol Hypertens 21: 258–263. 22388551 10.1097/MNH.0b013e3283522e7a

[2] Patrakka J, Tryggvason K (2010) Molecular make-up of the glomerular filtration barrier. Biochem Biophys Res Commun 396: 164–169. 20494132 10.1016/j.bbrc.2010.04.069

[3] Kashtan CE, Segal Y (2011) Genetic disorders of glomerular basement membranes. Nephron Clin Pract 118: c9–c18. 21071975 10.1159/000320876

[4] Khoshnoodi J, Pedchenko V, Hudson BG (2008) Mammalian collagen IV. Microsc Res Tech 71: 357–370. 18219669 10.1002/jemt.20564 PMC4788096

[5] Miner JH (2011) Organogenesis of the kidney glomerulus: Focus on the glomerular basement membrane. Organogenesis 7: 75–82. 21519194 10.4161/org.7.2.15275 PMC3142441

[6] Miner JH (1999) Renal basement membrane components. Kidney Int 56: 2016–2024. 10594777 10.1046/j.1523-1755.1999.00785.x

[7] Zenker M, Aigner T, Wendler O, Tralau T, Munterfering H, et al (2004) Human laminin beta2 deficiency causes congenital nephrosis with mesangial sclerosis and distinct eye abnormalities. Hum Mole Genet 13: 2625–2632. 10.1093/hmg/ddh284 15367484

[8] Cosgrove D, Meehan DT, Grunkemeyer JA, Kornak JM, Sayers R, et al (1996) Collagen COL4A3 knockout: a mouse model for autosomal Alport syndrome. Genes Dev 10: 2981–2992. 8956999 10.1101/gad.10.23.2981

[9] Miner JH, Sanes JR (1996) Molecular and functional defects in kidneys of mice lacking collagen alpha 3(IV): implications for Alport syndrome. J Cell Biol 135 (5) 1403–1413. 8947561 10.1083/jcb.135.5.1403 PMC2121079

[10] Cosgrove D, Kalluri R, Miner JH, Segal Y, Borza DB (2007) Choosing a mouse model to study the molecular pathobiology of Alport glomerulonephritis. Kidney Int 71: 615–618. 17290292 10.1038/sj.ki.5002115

[11] Cosgrove D, Rodgers K, Meehan D, Miller C, Bovard K, et al (2000) Integrin α1β1 and transforming growth factor-β1 play distinct roles in Alport glomerular pathogenesis and serve as dual targets for metabolic therapy. Am J Pathol 157: 1649–1659. 11073824 10.1016/s0002-9440(10)64802-x PMC1885718

[12] Kashtan CE, Kim Y, Lees GE, Thorner PS, Virtanen I, et al (2001) Abnormal glomerular basement membrane laminins in murine, canine, and human Alport syndrome: aberrant laminin alpha2 deposition is species independent. J Am Soc Nephrol 12: 252–260. 11158215 10.1681/ASN.V122252

[13] Abrahamson DR, Prettyman AC, Robert B, St John PL (2003) Laminin-1 reexpression in Alport mouse glomerular basement membranes. Kidney Int 63: 826–834. 12631063 10.1046/j.1523-1755.2003.00800.x

[14] Abrahamson DR, Hudson BG, Stroganova L, Borza DB, St John PL (2009) Cellular origins of type IV collagen networks in developing glomeruli. J Am Soc Nephrol 20: 1471–1479. 19423686 10.1681/ASN.2008101086 PMC2709682

[15] Putaala H, Soininen R, Kilpeläinen P, Wartiovaara J, Tryggvason K (2001) The murine nephrin gene is specifically expressed in kidney, brain and pancreas: inactivation of the gene leads to massive proteinuria and neonatal death. Hum Mol Genet 10: 1–8. 11136707 10.1093/hmg/10.1.1

[16] Roselli S, Heidet L, Sich M, Henger A, Kretzler M, et al (2004) Early glomerular filtration defect and severe renal disease in podocin-deficient mice. Mol Cell Biol 24: 550–560. 14701729 10.1128/MCB.24.2.550-560.2004 PMC343810

[17] Kalluri R, Shield CF, Todd P, Hudson BG, Neilson EG (1997) Isoform switching of type IV collagen is developmentally arrested in X-linked Alport syndrome leading to increased susceptibility of renal basement membranes to endoproteolysis. J Clin Invest 99: 2470–2478. 9153291 10.1172/JCI119431 PMC508088

[18] Meehan DT, Delimont D, Cheung L, Zallocchi M, Sansom SC, et al (2009) Biomechanical strain causes maladaptive gene regulation, contributing to Alport glomerular disease. Kidney Int 76: 968–976. 19710627 10.1038/ki.2009.324 PMC2780007

[19] Zeisberg M, Khurana M, Rao VH, Cosgrove D, Rougier JP, et al (2006) Stage-specific action of matrix metalloproteinases influences progressive hereditary kidney disease. PLoS Med 3: e100. 16509766 10.1371/journal.pmed.0030100 PMC1391977

[20] Wyss HM, Henderson JM, Byfield FJ, Bruggeman LA, Ding Y, et al (2011) Biophysical properties of normal and diseased renal glomeruli. Am J Physiol Cell Physiol 300: C397–C405. 21123730 10.1152/ajpcell.00438.2010 PMC3063968

[21] Abrahamson DR, Isom K, Roach E, Stroganova L, Zelenchuk A, et al (2007) Laminin compensation in collagen α3(IV) knockout (Alport) glomeruli contributes to permeability defects. J Am Soc Nephrol 18: 2465–2472. 17699809 10.1681/ASN.2007030328

[22] Drenckhahn D, Franke RP (1988) Ultrastructural organization of contractile and cytoskeletal proteins in glomerular podocytes of chicken, rat, and man. Lab Invest 59: 673–682. 3141719

[23] Yaoita E, Franke WW, Yamamoto T, Kawasaki K, Kihara I (1999) Identification of renal podocytes in multiple species: Higher vertebrates are vimentin positive/lower vertebrates are desmin positive. Histochem Cell Biol 111: 107–115. 10090571 10.1007/s004180050340

[24] Cortes P, Méndez M, Riser BL, Guérin CJ, Rodríguez-Barbero A, et al (2000) F-actin fiber distribution in glomerular cells: structural and functional implications. Kidney Int 58: 2452–61. 11115078 10.1046/j.1523-1755.2000.00428.x

[25] Wang R, St John PL, Kretzler M, Wiggins RC, Abrahamson DR (2000) Molecular cloning, expression, and distribution of glomerular epithelial protein 1 in developing mouse kidney. Kidney Int 57: 1847–18 59. 10792603 10.1046/j.1523-1755.2000.00034.x

[26] Mundel P, Heid HW, Mundel TM, Kruger M, Reiser J, et al (1997) Synaptopodin: an actin-associated protein in telencephalic dendrites and renal podocytes. J Cell Biol 139: 193–204. 9314539 10.1083/jcb.139.1.193 PMC2139823

[27] Helfand BT, Chang L, Goldman RD (2004) Intermediate filaments are dynamic and motile elements of cellular architecture. J Cell Sci 117: 133–141. 14676269 10.1242/jcs.00936

[28] Eriksson JE, Dechat T, Grin B, Helfand B, Mendez M, et al (2009) Introducing intermediate filaments: From discovery to disease. J Clin Invest 119: 1763–1771. 19587451 10.1172/JCI38339 PMC2701876

[29] Dechat T, Pfleghaar K, Sengupta K, Shimi T, Shumaker DK, et al (2008) Nuclear lamins: Major factors in the structural organization and function of the nucleus and chromatin. Genes Dev 22: 832–853. 18381888 10.1101/gad.1652708 PMC2732390

[30] Ivaska J, Pallari HM, Nevo J, Eriksson JE (2007) Novel functions of vimentin in cell adhesion, migration, and signaling. Exp Cell Res 313: 2050–2062. 17512929 10.1016/j.yexcr.2007.03.040

[31] Eriksson JE, He T, Trejo-Skalli AV, Harmala-Brasken AS, Hellman J, et al (2004) Specific in vivo phosphorylation sites determine the assembly dynamics of vimentin intermediate filaments. J Cell Sci 117: 919–932. 14762106 10.1242/jcs.00906

[32] Satelli A, Shulin L (2011) Vimentin in cancer and its potential as a molecular target for cancer therapy. Cell Mol Life Sci 68: 3033–3046. 21637948 10.1007/s00018-011-0735-1 PMC3162105

[33] Colucci-Guyon E, Portier MM, Dunia J, Paulin D, Pournin S, et al (1994) Mice lacking vimentin develop and reproduce without an obvious phenotype. Cell 79: 679–694. 7954832 10.1016/0092-8674(94)90553-3

[34] Colucci-Guton E, Gimenez M, Ribotta Y, Maurice T, Babinet C, et al (1999) Cerebellar defect and impaired motor coordination in mice lacking vimentin. Glia 25: 33–43. 9888296

[35] Eckes B, Colluci-Guyon E, Smola H, Nodder C, Babinet C, et al (2000) Impaired wound healing in embryonic and adult mice lacking vimentin. J Cell Sci 113: 2455–2462. 10852824 10.1242/jcs.113.13.2455

[36] Nieminen M, Henttinen T, Merinen M, Marttila-Ichihara F, Eriksson JE, et al (2006) Vimentin function in lymphocyte adhesion and transcellular migration. Nat Cell Biol 8: 156–162. 16429129 10.1038/ncb1355

[37] Holthofer H, Miettinen A, Lehto VP, Lehtonen E, Virtanen I (1984) Expression of vimentin and cytokeratin types of intermediate filament proteins in developing and adult human kidney. Lab Invest 50: 552–559. 6201675

[38] Stamenkovic I, Skalli O, Gabbiani G (1986) Distribution of intermediate filament proteins in normal and diseased human glomeruli. Am J Pathol 125: 465–475. 2432791 PMC1888470

[39] Zou J, Yaoita E, Watanabe Y, Yoshida Y, Nameta M, et al (2006) Upregulation of nestin, vimentin, and desmin in rat podocytes in response to injury. Virchows Arch 448: 485–492. 16418842 10.1007/s00428-005-0134-9

[40] Zou J, Chang TH, Chang H, Yaoita E, Yoshida Y, et al (2007) Time course of expression of intermediate filament protein vimentin, nestin, and desmin in rat renal glomerular injury. Chin Med J 120: 1203–1205. 17637254

[41] Shi S, Yu L, Chiu C, Sun Y, Chen J, et al (2008) Podocyte-selective deletion of dicer induces proteinuria and glomerulosclerosis. J Am Soc Nephrol 19: 2159–2169. 18776119 10.1681/ASN.2008030312 PMC2573016

[42] Ostalska-Nowicka D, Zachwieja J, Nowicki M, Witt M (2004) Expression of intermediate filaments of podocytes within nephrotic syndrome glomerulopathies in children. Histochem Cell Biol 121: 109–113. 14740224 10.1007/s00418-004-0619-7

[43] Perry J, Ho M, Viero S, Zheng K, Jacobs R, et al (2007) The intermediate filament nestin is highly expressed in normal human podocytes and podocytes in disease. Pediatr Dev Pathol 10: 369–382. 17929992 10.2350/06-11-0193.1

[44] Hynes RO (2002) Integrins: Bidirectional, allosteric signaling machines. Cell 110: 673–687. 12297042 10.1016/s0092-8674(02)00971-6

[45] Kreis S, Schönfeld HJ, Melchior C, Steiner B, Kieffer N (2005) The intermediate filament protein vimentin binds specifically to a recombinant integrin alpha2/beta1 cytoplasmic tail complex and co-localizes with native alpha2/beta1 in endothelial cell focal adhesions. Exp Cell Res 305 (1) 110–21. 15777792 10.1016/j.yexcr.2004.12.023

[46] Ivaska J, Vuoriluoto K, Huovinen T, Izawa I, Inagaki M, et al (2005) PKCε-mediated phosphorylation of vimentin controls integrin recycling and motility. EMBO J 24: 3834–3845. 16270034 10.1038/sj.emboj.7600847 PMC1283946

[47] Pozzi A, Zent R (2011) Extracellular matrix receptors in branched organs. Curr Opin Cell Biol 23: 547–553. 21561755 10.1016/j.ceb.2011.04.003 PMC3181278

[48] Kuhara T, Kagami S, Kuroda Y (1997) Expression of beta-1 integrins on activated mesangial cells in human glomerulonephritis. J Am Soc Nephrol 8: 1679–1687. 9355070 10.1681/ASN.V8111679

[49] Shikata K, Makino H, Moriaka S, Kashitani T, Hirata K, et al (1995) Distribution of extracellular receptors in various forms of glomerulonephritis. Am J Kidney Dis 25: 680–688. 7538261 10.1016/0272-6386(95)90542-1

[50] Chen X, Moeckel G, Morrow JD, Cosgrove D, Harris RC, et al (2004) Lack of integrin alpha 1 beta 1 leads to severe glomerulosclerosis after glomerular injury. Am J Pathol 165: 617–630. 15277235 10.1016/s0002-9440(10)63326-3 PMC1618576

[51] Zent R, Yan X, Su Y, Hudson BG, Borza DB, et al (2006) Glomerular injury is exacerbated in diabetic integrin alpha-1 null mice. Kidney Int 70: 460–470. 16775606 10.1038/sj.ki.5000359

[52] Mathew S, Chen X, Pozzi A, Zent R (2012) Integrins in renal development. Pediatr Nephrol 27: 891–900. 21603909 10.1007/s00467-011-1890-1

[53] Kreidberg JA, Donovan MJ, Goldstein SL, Renneke H, Shepherd K, et al (1996) Alpha 3 beta 1 integrin has a crucial role in kidney and lung organogenesis. Development 122: 3537–3547. 8951069 10.1242/dev.122.11.3537

[54] Sachs N, Kreft M, van den Bergh Weerman MA, Beynon AJ, Peters TA, et al (2006) Kidney failure in mice lacking the tetraspanin CD151. J Cell Biol 175: 33–39. 17015618 10.1083/jcb.200603073 PMC2064491

[55] Takemoto M, Asker N, Gerhardt H, Lundkvist A, Johansson BR, et al (2002) A new method for large scale isolation of kidney glomeruli from mice. Am J Pathol 161 (3) 799–805. 12213707 10.1016/S0002-9440(10)64239-3 PMC1867262

[56] Franco AT, Friedman DB, Nagy TA, Romero-Gallo J, Krishna U, Kendall A, et al (2009) Delineation of a carcinogenic Helicobacter pylori proteome. Mol Cell Proteomics 8: 1947–1958. 19470446 10.1074/mcp.M900139-MCP200 PMC2722763

[57] Yates JR, Eng JK, McCormack AL, Schieltz D (1995) Method to correlate tandem mass spectra of modified peptides to amino acid sequences in the protein database. Anal Chem 67: 1426–1436. 7741214 10.1021/ac00104a020

[58] Ma ZQ, Dasari S, Chambers MC, Litton MD, Sobecki SM, et al (2009) IDPicker 2.0: Improved protein assembly with high discrimination peptide identification filtering. J Proteome Res 8: 3872–3881. 19522537 10.1021/pr900360j PMC2810655

[59] Livak KJ, Schmittgen TD (2001) Analysis of relative gene expression data using real-time quantitative PCR and the 2(−Delta Delta C(T)) method. Methods 25: 402–408. 11846609 10.1006/meth.2001.1262

[60] Heidet L, Borza DB, Jouin M, Sich M, Mattei MG, et al (2003) A human-mouse chimera of the α3α4α5(IV) collagen promoter rescues the renal phenotype in Col4a3^−/−^ Alport mice. Am J Pathol 165: 1633–1644. 10.1016/s0002-9440(10)63520-1 PMC1868284 14507670

[61] Abrahamson DR, Irwin MH, St.John PL, Perry EW, Accavitti MA, et al (1989) Selective immunoreactivities of kidney basement membranes to monoclonal antibodies against laminin: localization of the end of the long arm and short arms to discrete microdomains. J Cell Biol 109: 3477–3491. 2480964 10.1083/jcb.109.6.3477 PMC2115970

[62] Abramoff MD, Magelhaes PJ, Ram SJ (2004) Image Processing with ImageJ. Biophotonics Int 11: 36–42.

